# Prophylactic *Faecalibacterium prausnitzii* treatment prevents the acute breakdown of colonic epithelial barrier in a preclinical model of pelvic radiation disease

**DOI:** 10.1080/19490976.2020.1812867

**Published:** 2020-09-28

**Authors:** Alexia Lapiere, Mallia Geiger, Véronique Robert, Christelle Demarquay, Sandrine Auger, Sead Chadi, Mohamedamine Benadjaoud, Gabriel Fernandes, Fabien Milliat, Philippe Langella, Marc Benderitter, Jean-Marc Chatel, Alexandra Sémont

**Affiliations:** a Department of RAdiobiology and Regenerative MEDicine (SERAMED), Laboratory of MEDical Radiobiology (Lrmed), Institute for Radiological Protection and Nuclear Safety (IRSN), Fontenay-aux-Roses, France; b INRAE, AgroParisTech, Micalis Institute, Paris-Saclay University, Jouy-en-Josas, France; c Department of RAdiobiology and Regenerative MEDicine (SERAMED), Institute for Radiological Protection and Nuclear Safety (IRSN), Fontenay-aux-Roses, France; d René Rachou Institute, Oswaldo Cruz Foundation, Belo Horizonte, MG, Brazil

**Keywords:** *Faecalibacterium prausnitzii*, prophylactic treatment, pelvic radiation disease, colonic epithelial barrier, inflammation, tuft cells

## Abstract

Every year, millions of people around the world benefit from radiation therapy to treat cancers localized in the pelvic area. Damage to healthy tissue in the radiation field can cause undesirable toxic effects leading to gastrointestinal complications called pelvic radiation disease. A change in the composition and/or function of the microbiota could contribute to radiation-induced gastrointestinal toxicity. In this study, we tested the prophylactic effect of a new generation of probiotic like *Faecalibacterium prausnitzii* (*F. prausnitzii*) on acute radiation-induced colonic lesions. Experiments were carried out in a preclinical model of pelvic radiation disease. Rats were locally irradiated at 29 Gray in the colon resulting in colonic epithelial barrier rupture. Three days before the irradiation and up to 3 d after the irradiation, the *F. prausnitzii* A2-165 strain was administered daily (intragastrically) to test its putative protective effects. Results showed that prophylactic *F. prausnitzii* treatment limits radiation-induced para-cellular hyperpermeability, as well as the infiltration of neutrophils (MPO+ cells) in the colonic mucosa. Moreover, *F. prausnitzii* treatment reduced the severity of the morphological change of crypts, but also preserved the pool of Sox-9+ stem/progenitor cells, the proliferating epithelial PCNA+ crypt cells and the Dclk1+/IL-25+ differentiated epithelial tuft cells. The benefit of *F. prausnitzii* was associated with increased production of IL-18 by colonic crypt epithelial cells. Thus, *F. prausnitzii* treatment protected the epithelial colonic barrier from colorectal irradiation. New-generation probiotics may be promising prophylactic treatments to reduce acute side effects in patients treated with radiation therapy and may improve their quality of life.

## Introduction

Cancer is the second leading cause of death globally. Radiation therapy, an established part of treatment of both primary and recurrent pelvic malignancies, is used in almost 50% of cases and concerns millions of people worldwide each year. Improvement in treatment in the last decades has led to a threefold increase in the number of cancer survivors over the last 30 y. The efficacy of radiation therapy requires an optimal compromise between tumor control and healthy, normal tissue tolerance; this is referred to as the benefit/risk ratio. Following radiation therapy, healthy tissue around the tumor can be damaged, initiating in 90% of patients acute, and in 5% to 10% of patients, chronic side effects. A high incidence of acute symptoms is reported during or shortly after the end of the radiation therapy. In some cases, depending on dose, volume, as well as individual susceptibilities, severe acute symptoms may result in treatment suspension, and thus failure of tumor control. Moreover, more than 10 y after the end of the treatment, patients may exhibit persistent overlapping symptoms (diarrhea, constipation, bloating, pain), resulting from multiple visceral organ dysfunctions. All these symptoms were recognized in 2010 as a new disease entity called “Pelvic Radiation Disease” (PRD).^1^

Digestive mucosa constitutes a physical barrier, the first line of defense against pathogen infiltration in the organism. Due to the high proliferative capacity of crypt epithelial stem/progenitor cells, colonic mucosa is highly radiosensitive. After local radiation exposure, a complex cascade of biological effects, i.e. from damage of multiple biomolecules to cell phenotype modification and/or death, may induce disruption of barrier integrity. Colonic barrier disruption may drive bacterial translocation and likely the maintenance and/or exacerbation of mucosal inflammation.^2^ After local radiation exposure, in the absence of acute immune response control, chronic inflammation could lead to persistent and irreversible damage. So, protecting the colonic mucosa from radiation toxicity and therefore from the progression of inflammation might result in the alleviation of acute and chronic side effects and should improve the patient’s quality of life and/or reduce the risk of life-threatening conditions.

In 2014, Andreyev suggested that intestinal microbiota could be implicated in the initiation and/or maintenance of radiation-induced intestinal toxicity.^3^ In patients receiving pelvic radiation therapy, there is a loss of microbial diversity, which is more pronounced in those with side effects like diarrhea. Among observed abundance variation, bacteria belonging to the genus Faecalibacterium were reduced.^4,5^ *Faecalibacterium prausnitzii* (*F. prausnitzii*) is one of the most abundant anaerobic bacteria in the human colon (up to 5%) and so far the only member of the Faecalibacterium genus. Preclinical experiments performed in animal models of inflammatory bowel disease have previously demonstrated the therapeutic benefit of prophylactic treatment with *F. prausnitzii*, which reduces colonic damage and leads to increased survival of animals.^6-10^ Earlier studies demonstrated an ability of *F. prausnitzii* to reduce histological and functional colonic barrier impairment. The beneficial effects of *F. prausnitzii* were associated with its tendency to counterbalance dysbiosis and with local or systemic anti-inflammatory effects.^6,7,9,11,12^ Sokol et al.^6^ reported that live *F. prausnitzii* administration prevents an acute TNBS-induced increase in the pro-inflammatory cytokine IL-12, but only tends to increase the anti-inflammatory cytokine IL-10 in the colon. Some other pro-inflammatory cytokines are also reduced by live *F. prausnitzii* administration in the colon of mice with chronic low-grade inflammation: TNF-α, IFN-γ and IL-6.^9,10^ While these studies have outlined a protective effect of *F. prausnitzii* treatment on the colonic mucosa, probably in part via management of the inflammatory response, the overall effect of *F. prausnitzii* on the proliferation and self-renewal of crypt epithelial cells remains unknown.

In this study, we made the assumption that *F. prausnitzii* administration might preserve colonic mucosa from radiation damage by controlling the immune response. Our objectives were to test the effectiveness of prophylactic *F. prausnitzii* treatment on radiation-induced colonic mucosal barrier breakdown. We analyzed various parameters to assess the maintenance and/or disruption of colonic mucosal barrier integrity, such as the histological structure and self-renewal of the colonic epithelium, para- and trans-cellular permeability, microbiota composition/diversity and the inflammatory process. In a context of radiation-induced colonic mucosal toxicity and dysfunction, we found that prophylactic *F. prausnitzii* treatment had protective effects. We demonstrated that this effect is associated with the ability of *F. prausnitzii* to prevent acute para-cellular permeability and therefore the nonspecific immune response. Our results also suggested for the first time that the therapeutic benefit of *F. prausnitzii* might also follow activation of protected epithelial cells and underlined the probable involvement of differentiated epithelial cells, principally tuft cells.

## Materials and methods

### Ethics statement

All experiments were performed in compliance with the Guide for the Care and Use of Laboratory Animal as published by the French regulations for animal experiments (Ministry of Agriculture Order No. B92-032-01, 2006) and with European Directives (86/609/CEE) and were approved by the local ethics committee of our institute (permit number P16-07).

### Animals

Male SD (Sprague Dawley) rats (Janvier SA, Le Genest St Isle, France) weighing 250–300 g were housed in standard conditions for a minimum of 1 week before experimentation. They were allowed free access to water and fed standard pellets.

### Bacterial strains and growth conditions

*Faecalibacterium prausnitzii* strain A2-165 (DSMZ collection, Braunschweig, Germany) (DSM No 17677) was grown in LYBHI medium (brain-heart infusion medium supplemented with 0.5% yeast extract) (Difco, Detroit, USA) supplemented with 1 mg/mL cellobiose (Sigma-Aldrich Chemie GmbH, Buchs, Switzerland), 1 mg/mL maltose (Sigma-Aldrich), and 0.5 mg/mL cysteine (Sigma-Aldrich) at 37°C in an anaerobic chamber (90% N_2_, 5% CO,_2_ and 5% H_2_). Bacteria were harvested by centrifugation, washed in PBS, re-suspended in PBS containing 20% glycerold stored at −80°C.

### Irradiation protocol

Rats were anesthetized by isoflurane inhalation and a single 29 Gray (Gy) dose of irradiation was delivered by a medical accelerator (Alphée) through a 2x3cm window centered on the colorectal region. Alphée is an accelerator-type radiation source (maximal energy is 4 MeV with an average energy of about 1.5 MeV; 30 kA).

### Experimental design

Two groups of rats were submitted to colorectal irradiation at a single 29 Gy dose and one group was not irradiated (control group). The irradiated rats received prophylactic treatment with *F prausnizii* or received only vehicle. Non-irradiated group received vehicle. Three days before irradiation, the day of irradiation, and 2 d after irradiation, 1.10^9^ CFU of bacteria or PBS was intragastrically administered once daily. Rats were sacrificed, 6 h, 3 d after irradiation. The study groups were as follows: Control group (Ctrl PBS): non-irradiated and PBS-treated rats, Irradiated group (IRR PBS): irradiated and PBS-treated rats, and -*F. prausnitzii* treated group (IRR Fprau): irradiated and *F. prausnitzii* -treated rats.

In the first batch of the experiment, the colonic barrier integrity was analyzed on colonic sections by histological analysis, immunohistochemistry/immunofluorescence staining and TUNEL assay, 6 h or 3 d after irradiation. One independent experiment for each time of sacrifice (N = 1, 5 animals per groups) was performed (8 to 20 independent colonic sections per rats were analyzed) and structural damages (morphological crypt atypia or crypt loss; HES staining), epithelium renewal ability (TUNEL and PCNA-positive crypt epithelial cells in apoptosis or proliferation, respectively, SOX9-positive progenitor/stem cells and DclK1-positive Tuft cells) and inflammatory process (mucosal infiltration of MPO-positive neutrophils and CD68-positive macrophages and IL-18-positive crypt epithelial cells) were assessed.

In a second batch of the experiment, the colonic barrier integrity was analyzed by measuring *ex vivo* colonic trans- and para-cellular colonic permeability. Three independent experiments were performed (N = 3, between 8 and 12 animals per group for each experiment).

In a third batch of the experiment, 3 d after irradiation, fecal microbiota composition by the V3-V4 hyper-variable region of the 16SrRNA sequencing and also mucosal cytokine concentration using ELISA bio-plex cytokine assay (23 cytokines including IL-18) were assessed. One independent experiment was performed (N = 1, between 9 and 12 animals per group).

In a fourth batch of the experiment, the colonic barrier integrity was analyzed 7 d after irradiation by histological analysis performed on colonic sections stained with HES (See Supplementary materials and methods). One independent experiment was performed (N = 1, 8 animals per groups, 20 independent colonic sections per rats were analyzed) and the severity of colonic mucosal ulceration was measured.

### Histological and damage analysis

Animals were sacrificed by isoflurane inhalation 3 d after colorectal irradiation. Distal colons were removed, fixed in 4% formaldehyde, cross-sectioned in four equal pieces and embedded in paraffin. Paraffin-embedded colons were cut on a rotary microtome (Leica Microsystems AG, Wetzlar, Germany) into serial circular sections of 5 µm, in 5 series spaced by 250 µm and stained with hematoxylin-eosin-saffron (HES). Each slide was composed of four circular sections of the cross-sectioned colon representative of lesion along the colon. The severity of histological damage of the mucosa on each colonic circular section was assessed using a lesion score with the Histolab software (Microvision Instruments, Lisses, France). Determination of the injury score was based on (1) inflammatory infiltration (modest inflammatory infiltration at the base of the crypt leading to slight crypt detachment from the lamina propria; 0< score<1, or inflammatory infiltration in all the lamina propria; 1< score<2), (2) epithelial damage (crypt morphology atypia 0< score<1, and loss of epithelial crypts (1< score<2) or both (1 + 2; in this case graduation of the injury was: score 0 = null; 0< score<1 = slight; 1< score<2 = moderate and score>2 = severe). The injury score was calculated for each animal group. Morphometric analysis of crypt depth was also performed on HES-stained slides.

### Immunohistochemistry and immunofluorescence

For immunohistochemistry, sections were deparaffinized and hydrated. Tissue sections were treated with 0.1% Triton X-100 (Sigma-Aldrich) in PBS 1x (Life Technologies) at room temperature (RT) for 10 min. Then, endogenous peroxidases were inhibited by incubation with 3% H_2_O_2_ in methanol at RT for 10 min. Citrate buffer (10 mM, pH6) (Zytomed) was used as an antigen retrieval solution; incubation lasted 15 min at 350 W. After saturation (X0909, Dako), rabbit anti-SOX9 (ab185230, Abcam, 0.803–0.971 mg/mL, for stem/progenitor epithelial cell staining) diluted to 1/2000 and rabbit anti-MPO (IMG-803971, Abcam, for neutrophil staining) diluted to 1/75 was applied for 16 h at 4°C. After saturation (X0909, Dako), mouse anti-PCNA antibody diluted to 1/10000 (M0879 Dako, 525 µg/mL, for proliferating cell nuclear staining), and mouse anti-CD68 (MCA341R, AbDserotec, for resident macrophage staining) diluted to 1/200 was applied for 1 h at 37°C. Sections were then incubated for 30 min at RT with, respectively, anti-rabbit-HRP Ig (D13-110, GBI Labs) or anti-mouse-HRP Ig (D55-18, GBI Labs). Staining was developed with HRP Green (ZUC 070–100, Zytomed). The sections were then counterstained with nuclear fast red (H3403, VectorLabs), dehydrated, and mounted. The negative control was assessed with rabbit and mouse IgG (Dako, X0931) instead of the analyzed primary antibodies.

For immunofluorescence, sections were deparaffinized and hydrated, and incubated with 3% H_2_O_2_ in methanol followed by an incubation in citrate buffer. After saturation (X0909, Dako), rabbit anti-Dclk1 (ab109029, Abcam, for tuft cell staining) diluted to 1/300 or rabbit IL-25 (IL-17E, ab108530, Abcam) diluted to 1/500 was applied for 16 h at 4°C. After saturation (X0909, Dako), rabbit anti-IL-18 (HPA003980, Atlas antibodies) diluted to 1/50 was applied for 1 h at 37°C. For DclK1 or IL-18, sections were then incubated with donkey anti-rabbit Alexa Fluor 488 (A21206, Invitrogen) diluted to 1/200 for 1 h at RT. For IL-25, sections were then incubated with goat anti-rabbit Alexa Fluor 568 (A11011, life technologies) diluted to 1/200 for 1 h at RT. For Dclk1/IL25 double staining, immunostaining of IL25 and DclK1 were performed one by one starting by IL25. Slides were counterstained with DAPI and mounted with Vectashield (H-1000, Vector). The negative control was assessed with rabbit IgG (Dako, X0903) instead of the analyzed primary antibodies.

### Ex vivo para- and trans-cellular permeability measurement in using chambers

Three days after colorectal irradiation rats were sacrificed. Immediately after sacrifice, distal colons were removed, cut along the mesenteric border and mounted in Ussing chambers (Corning Costar Corporation, Harvard Apparatus, France) with a flux area of 1.78 cm2. Colonic cellular permeability to small and large molecules was measured through the mucosal-to-serosal passage of fluoroisothiocyanate (FITC)-Dextran 4 kDa (FD4; Sigma-Aldrich, Saint Quentin Fallavier, France) and intact horseradish peroxidase 44 kDa (HRP; Sigma-Aldrich, Saint Quentin Fallavier, France) respectively, added simultaneously in the mucosal compartment. After 20 min of equilibration, 600 µL of buffer solution on the mucosal side was replaced by 300 µL of FD4 (2.2 mg/mL) and 300 µL of HRP (0,4 mg/ml). After 1-h incubation, colonic permeability to FD4 was determined by measuring the fluorescence intensity at 485 nm/525 nm using an automatic Infinite M200 microplate reader (Tecan, Lyon, France). Cellular permeability to intact HRP was determined by enzymatic assay^13^ for specific HRP activity found in the serosal and mucosal compartment using a microplate reader (Tecan). Permeability was calculated as the ratio of flux/concentration, as previously described^14^ and expressed in cm/second. Finally, comparison between groups was assessed by calculating relative percentage according to Ctrl PBS permeability.

### Colonic protein extraction and Bio-Plex cytokine assay

Colon mucosa proteins were extracted following the instructions for the Bio-Plex Cell Lysis kit (171304011, Bio-Rad). Colonic concentrations of 23 cytokines and chemokines (IL-1α, IL-1β, IL-2, IL-4, IL-5, IL-6, IL-7, IL-10, IL-12, IL-13, IL-17, IL-18, IFN-γ, TNF-α, VEGF, G-CSF, M-CSF, GM-CSF, GRO/KC, MCP-1, MIP-3α, MIP-1α and RANTES) were measured in extracts from colon tissue using the Bio-Plex Pro Rat Cytokine 23 (12005641, Bio-Rad) according to the manufacturer’s instructions. This Bio-Plex 200 system uses fluorescently dyed beads to detect different types of molecules in a single well of a 96-microwell plate, requiring low sample volumes. Briefly, premixed beads coated with target antibodies (50 µL) were added to each well and washed twice with Bio-Plex wash buffer. Standards (eight concentrations ranging from 32000 to 1.95 pg/mL) and samples (50 µL) were then added to the wells, followed by shaking at 1100 rpm for 30 sec and incubation for 30 min with shaking at 300 rpm at RT. Wells were then washed 3 times with Bio-Plex wash buffer, and premixed detection antibody (25 µL) was added to the wells. This was followed by shaking at 1100 rpm for 30 sec and incubation for 30 min with shaking at 300 rpm at RT. Wells were again washed 3 times with Bio-Plex wash buffer and streptavidin-PE (25 µL) was added to the wells. Incubation was for 10 min with shaking at 300 rpm. Wells were washed 3 times with Bio-Plex wash buffer and the beads were resuspended in 125 µL of Bio-Plex assay buffer. With this technology, relevant inflammatory cytokines could be detected in a single run. Dyed beads were read on the Bio-Plex analyzer. One laser classified the beads and determines the cytokine being detected, and a second laser determined the magnitude of the phycoerythrin-derived signal, which is in direct proportion to the amount of molecule bound. Cytokine concentrations were derived by interpolating the measured fluorescence intensities to standard curves and by correcting using the corresponding dilution factor employed to achieve the minimum volume for analysis. Bio-Plex Manager software (Bio-Rad) was employed to calculate cytokine concentrations. To avoid inter-assay variations, all samples were analyzed with the same kit on the same day.

### Fecal microbiota analysis

Total bacterial DNA was extracted from the fecal samples according to the protocol described in Godon et al.^15^ DNA concentration, purity, and integrity were determined using a NanoDrop instrument. The size distribution of the extracted DNA estimated by 1% agarose gel electrophoresis showed that most of the DNA was high molecular weight (>20 kb) with no significant shearing. These observations suggest that the extracted DNA was of good quality and suitable for downstream processing.

The V3-V4 hyper-variable region of the 16S rRNA gene was amplified with the primers MSQ-16SV3F (CTTTCCCTACACGACGCTCTTCCGATCTACGGRAGGCWGCAG) and MSQ-16SV4R (GGAGTTCAGACGTGTGCTCTTCCGATCTTACCAGGGTATCTAATCCT). The PCR reactions were performed using 10 ng of extracted DNA, 0.5 μM primer, 0.2 mM dNTP, and 0.5 U of the DNA-free Taq-polymerase, MolTaq 16S DNA Polymerase (Molzym). The amplifications were carried out using the following profile: 1 cycle at 94°C for 60 s, followed by 30 cycles at 94°C for 60 s, 65°C for 60 s, 72°C for 60 s, and finishing with a step at 72°C for 10 min. The resulting PCR products were purified and sent to the @BRIDGe platform (INRA, Jouy-en-Josas) for sequencing using Illumina MiSeq technology.

Sequences were analyzed using the Galaxy-supported pipeline FROGS to produce abundance tables of Operational Taxonomic Units (OTUs) and their taxonomic affiliation. The successive steps involved de-noising and clustering of the sequence into OTUs using SWARM; chimera removal using VSEARCH; and taxonomic affiliation for each OTU using RDP Classifier on the SILVA SSU 123 database. Statistical analyses were performed using “R” language and environment version 3.2.3. α- and β-diversity measurements and analysis of the differences in OTUs between samples was performed using the add-on package “Phyloseq”^16^

### Statistics

All data are presented as means (SEM). Statistical analyses were performed using Graph Pad Prism 5.0 (GraphPad, San Diego, CA). Statistical significance was determined using one-way ANOVA followed by Tukey’s posttest for multiple groups, or a t-test when changes were compared between two groups. Statistical significance was set at *p* <.05.

Differences between the cytokine concentrations were analyzed using generalized least squares (GLS) models with group as nominal covariate (Controls, irradiated and Irradiated+Fprau). The GLS function of the nlme R package offers a variety of variance structure specifications to handle violations of the variance homogeneity assumption in order to obtain correct standard errors and statistical significance. In each case, the selection of the optimal model was conducted using the likelihood ratio for nested models and the AIC otherwise. The significant level was fixed at *p* < .05 after Benjamini–Hochberg discovery rate (FDR) correction.

## Results

### Prophylactic F. prausnitzii treatment reduces radiation-induced histological damage to the colonic epithelium

**Figure 1.**
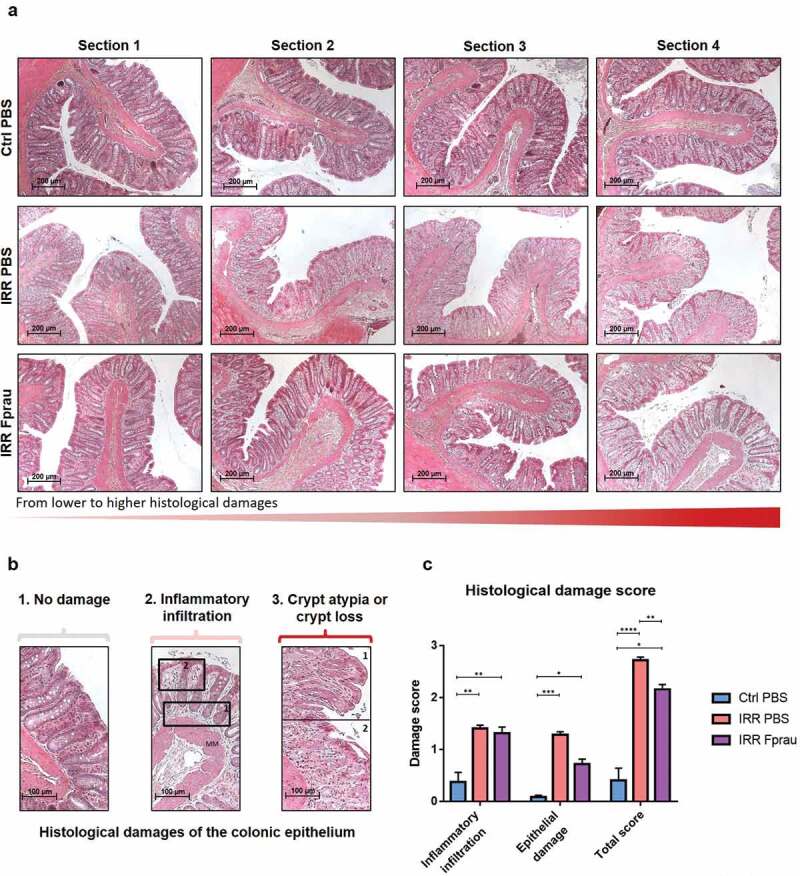
Effect of prophylactic *F. prausnitzii* treatment on 29 Gy colorectal irradiation-induced histological damage to the colonic mucosa at 3 d.

The colonic epithelium forms a selective barrier against the penetration of luminal contents and then commensal and pathogen bacterial translocation. Physical barrier integrity of the colon was investigated in scoring structural damage of the colonic epithelium on histology slides (Figure 1). In the control group, the histological damage score of the colonic epithelium was 0.40 ± 0.23 coming from rare zone with inflammatory infiltration and slight crypt detachment from the muscularis mucosa. Three days after colorectal irradiation, the score reached 2.72 ± 0.06 (*p* < .0001 vsvsvs control rats) corresponding to an increase in the number of sections with crypt morphological atypia (disorganized and deformed colonic epithelial crypts) or crypt loss but also with inflammatory infiltration in all the lamina propria. Compared to rats subjected to irradiation alone, in those pre-treated with *F. prausnitzii* we showed a significant reduction in the severity of the histological damage score (2.15 ± 0.09 in irradiated and *F. prausnitzii* treatment *vs* 2.71 ± 0.06 in irradiated rats, *p* = .0013). Histological images and the scoring of parameters individually (Figure 1(c)) show that an increased score in irradiated rats treated by *F prausnitzii* in comparison with control rats is mostly dependent on modest inflammatory infiltration leading to slight crypt detachment from muscularis mucosa but infrequently dependent of crypt morphological atypia. So, *F prausnitzii* seems to maintain normal crypt morphology. Our results demonstrate an ability of *F. prausnitzii* to partially protect the structural integrity of the colonic mucosa from colorectal irradiation and suggest it has the potential to preserve the epithelium as a physical barrier against bacterial translocation. To support this assumption the following experiments looked at how *F. prausnitzii* can regulate the function of the colonic epithelial barrier.

(a-b) Colonic sections were stained with hematoxylin, eosin and saffron (HES). (a) Sections show histological structure change of the colonic mucosa in one representative rat (4 representative sections/rat) of each group, the control group, the irradiated group and the irradiated and *F. prausnitzii*-treated group (Scale bars 200 µm). (b) Representative pictures showing parameters considered for histological scoring. (b1) No damage, (b2) inflammatory infiltration with a score graduation between 0 and 1 for modest inflammatory infiltration leading to slight crypt detachment from the muscularis mucosa (MM, rectangle 1) or between 1 and 2 for inflammatory infiltration observed in all the lamina propria area (rectangle 2) and (b3) epithelial damages with a score graduation between 0 and 1 for crypt morphological atypia characterized by disorganized and deformed epithelial crypts (rectangle 1) or between 1 and 2 for crypt loss (rectangle 2). Scale bars 100 µm (c) Histogram represents means±SEM of inflammatory infiltration score, epithelial damage score or total scoring including inflammatory infiltration score and epithelial damage score (for total scoring graduation of the injury was: score 0 = null; 0< score<1 = slight; 1< score<2 = moderate and score>2 = severe) quantified in control animals, in irradiated animals or in irradiated and *F. prausnitzii*-treated animals. Analyses were performed on 20 sections per animal and 5 animals were used per group (N = 1). Ctrl = Control, IRR = Irradiated, Fprau = *F. prausnitzii*. Error bars represent SEM, *p <.05, **p <.01, ***p <.001, ****p <.0001.

### Prophylactic F. prausnitzii treatment prevents radiation-induced disruption of the function of the colonic epithelial barrier

**Figure 2.**
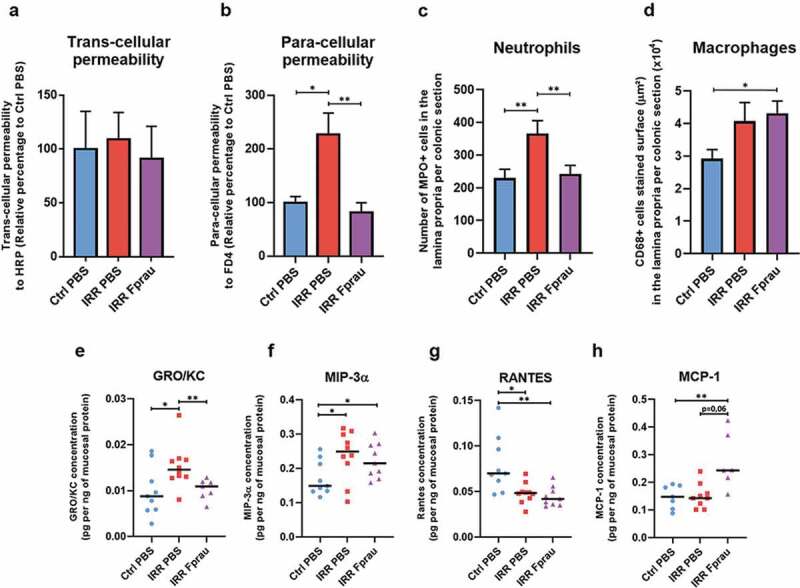
Effect of prophylactic *F. prausnitzii* treatment on 29 Gy colorectal irradiation-induced epithelial barrier breakdown at 3 d.

Low trans- and/or para-cellular permeability is a primordial index of the functional integrity of the colonic barrier. In this study, *ex vivo* colonic permeability using Ussing Chamber systems to measure trans-cellular permeability to intact horseradish peroxidase (HRP, 44KDa) and paracellular permeability to fluorescein isothiocyanate-dextran 4 (FD4, 4KDa) were quantified. At 3 d, colorectal irradiation in rats pre-treated with *F. prausnitzii* did not change trans-cellular permeability to HRP (Figure 2(a)). At the same time after colorectal irradiation, we found a 2.3-fold increase in paracellular permeability to FD4 (Figure 2(b), *p* < .05), neutrophil infiltration, as demonstrated by the increased number of MPO-positive neutrophils and expression of GRO/KC (a specific neutrophil chemo-attractant) in the colonic mucosa (Figure 2(c)¸2(e)), *p* < .01, *p* < .05, respectively), and probably antimicrobial activity, as suggested by increased expression of macrophage inflammatory protein 3α(MIP3α) by mucosal cells (Figure 2(f)), *p* < .05). Altogether, these findings point to colonic barrier breakdown 3 d after colorectal irradiation. Pre-treatment with *F. prausnitzii* protected rats from acute radiation-induced para-cellular hyperpermeability. Permeability values in irradiated rats pre-treated with *F. prausnitzii* remained close to basal level (Figure 2(b)). *F. prausnitzii* also prevented radiation-induced neutrophil infiltration, as shown by control levels of MPO-positive neutrophils and GRO/KC expression in the colonic mucosa after *F. prausnitzii* administration (Figure 2(c), 2(e)). MIP3α, remained elevated in irradiated rats pre-treated with *F. prausnitzii* (Figure 2(f)), *p* < .05).

(a-b) Measurement of *ex vivo* colonic trans- and para-cellular permeability to HRP or FD4, respectively, using Ussing chambers. Each value represents the average of two independent measurements per animal (15–20 animals per group) and is for three independently repeated experiments (N = 3). (c) Immunohistological quantitative analysis of neutrophil infiltration in the colonic lamina propria. Colonic sections were stained by myeloperoxidase (MPO) antibody. The number of MPO-positive cells was quantified in the lamina propria and per colonic section. (d) Immunohistological quantitative analysis of resident macrophages in the colonic lamina propria. Colonic sections were stained by CD68 antibody. The area in μm^2^ of CD68-positive cells stained was quantified in the lamina propria and per colonic section. For c and d, the histograms represent the average of eight independent measurements per animals (five animals per group (N = 1)). Ctrl = Controls, IRR = Irradiated, Fprau = *F. prausnitzii*. Error bars represent SEM. *p <.05, **p <.01. The graphs (e,f,g,h) represent the expression in the colonic mucosa of the chemokines GRO-KC, MIP-3α, RANTES and MCP-1 in control animals, irradiated animals or irradiated and *F. prausnitzii*-treated animals. ELISA multiplex assays were performed at least in duplicate per animal and 9 to 10 animals were used per group (N = 1). Ctrl = Controls, IRR = Irradiated, Fprau = *F. prausnitzii*. Error bars represent SEM, *p <.05, **p <.01.

### Prophylactic F. prausnitzii treatment increases resident macrophages in the colonic mucosa of rats subject to colorectal irradiation

Pre-treatment of irradiated rats with *F. prausnitzii* significantly increased CD68-positive resident macrophages in the colonic mucosa by 47.60%, while in rats not pretreated there was only a trend of increased macrophages (Figure 2(d)). Resident macrophages of the colon do not proliferate and appear to lack receptor-mediated chemotactic activity. Recruitment of circulating precursor monocytes seems to be the major source of cells that increase the number of resident macrophages after *F. prausnitzii* treatment. The chemokine RANTES plays a role in immune cell recruitment as monocytes, mast cells and lymphocytes, but its expression was reduced in rats not pre-treated and in rats pre-treated with *F. prausnitzii* compared to control rats (Figure 2(g)), *p* < .05, *p* < .01, respectively). Nevertheless, a borderline significant (*p* = .06) increase in MCP-1 in the colonic mucosa is suggestive of monocyte recruitment (Figure 2(h)). Our results also suggest that potential-recruited monocytes could be differentiating into macrophages, as the radiation-increased MIP-3αlevel in the colonic mucosa remained high in rats receiving *F. prausnitzii* treatment^17^ (Figure 2(f)).

### Colorectal irradiation with or without F. prausnitzii treatment doesn’t modify gut microbiota

**Figure 3.**
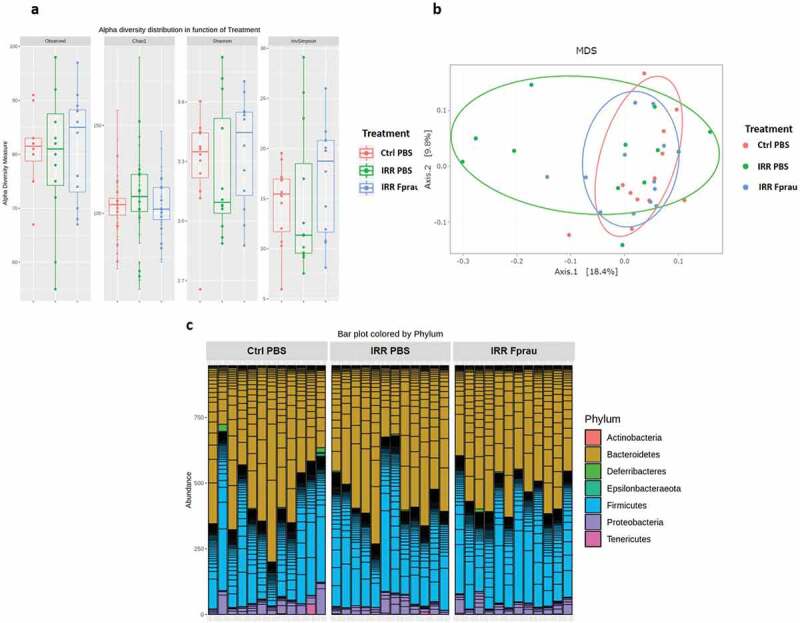
Microbiota composition analysis after 29 Gy colorectal irradiation in rats treated or not by *F. prausnitzii.*

We investigated whether colorectal irradiation with or without pre-treatment by *F. prausnitzii* involved modifications of the gut microbiota by analyzing the intestinal bacterial composition of the different experimental groups (Figure 3). We found no differences between groups in α-diversity, which measures the taxonomic richness of the fecal microbiota communities (Figure 3(a)). In addition, β-diversity analysis, which measures the degree of similarity between the gut microbial communities, revealed no clustering of the rats according to the *F. prausnitzii* or PBS pre-treatments before irradiation (Figure 3(b)). Bacteria composition analysis at the phyla level showed no differences between the groups (Figure 3(c)).

(a).16S rRNA-sequencing-based alpha diversity analysis, measured by Observed, Chao1, Shannon and invSimpson index in control, irradiated and *F. prausnitzii*-irradiated animals. (b) Principal Coordinates of analysis of all animals, confidence ellipses appear around each group. (c) Bacteria phyla analysis in all animals. Twelve animals were used per group for these analyses (N = 1). Ctrl = Controls, IRR = Irradiated, Fprau = *F. prausnitzii*.

### Prophylactic F. prausnitzii treatment increases the production of the cytokine IL-18 by crypt epithelial cells of irradiated rats

**Figure 4.**
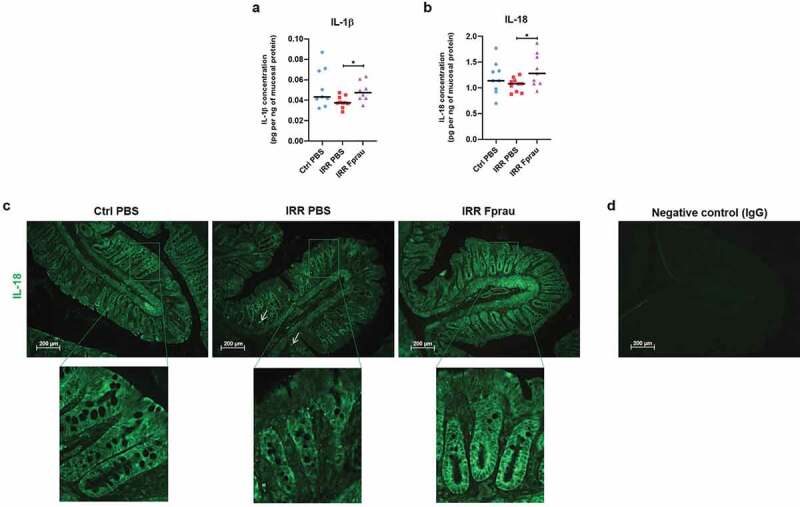
Effect of prophylactic *F. prausnitzii* treatment on IL-1β and IL-18 production by colonic mucosal cells 3 d after 29 Gy colorectal irradiation.

To study the acute and local inflammatory response to colorectal irradiation and to shed light on the mechanisms involved in the ability of *F. prausnitzii* to protect the colonic barrier from radiation toxicity, we assessed the mucosal profile and concentration of 17 cytokines (IL-1α, IL-1β, IL-2, IL-4, IL-5, IL-6, IL-7, IL-10, IL-12, IL-13, IL-17, IL-18, IFN-γ, TNF-α, G-CSF, M, CSF and GM-CSF) using a Bio-Plex assay (Figure 4(a-b), S1). At 3 d, colorectal irradiation did not change the expression level of any measured cytokines from the colonic mucosa. Nevertheless, in rats receiving prophylactic *F. prausnitzii* treatment, colorectal irradiation induced up-regulation of pro-inflammatory cytokines IL-1β and IL-18 by mucosal cells (Figure 4(a-b), irradiated rats *vs* irradiated and *F. prausnitzii* treated rats *p* < .05). As increased levels of IL-1β and IL-18 cytokines were not seen after irradiation alone (Figure 4(a-b)) or after bacteria administration alone (data not shown), it is likely that *F. prausnitzii* treatment and radiation exposure induced synergic effects on the production of these cytokines by colonic mucosal cells. In the colonic mucosa, IL-18 is expressed in a wide range of immune cells like macrophages as well as in epithelial cells. Immunohistochemistry on colonic tissue slides was performed to study the cellular origin of IL-18 production after *F. prausnitzii* treatment (Figure 4(c-d)). Even though we observed immunostaining of IL-18 in some immune cells localized in the lamina propria of the colonic mucosa (Figure 4(c), arrow), epithelial cells were the major source of its production. In control rats, we observed cytoplasmic immunostaining of IL-18 in the entire array of crypt epithelial cells. At 3 d, colorectal irradiation slightly reduced IL-18 production and immunostaining was more diffuse in the epithelial cytoplasm. In comparison with control rats, pre-treatment of irradiated rats with *F. prausnitzii* increased IL-18 production by epithelial cells, mostly those localized at the base of the crypt, suggesting an enhancement of basal production of IL-18 in the progenitor/stem cell compartment of the mucosa. We then analyzed whether increased IL-18 production by epithelial cells at the crypt base was associated with regulation of stem/progenitor cells.

(a, b) Graphs representing expression in the colonic mucosa of IL-1β and IL-18 in control animals, irradiated animals, or irradiated and *F. prausnitzii*-treated animals. ELISA multiplex assays were performed at least in duplicate per animal and 9 to 10 animals were used per group (N = 1). Error bars represent SEM, *p <.05. (c) Representative pictures of immunofluorescence staining of colonic sections by IL-18 antibody (green) in control animals, irradiated animals (arrows show IL-18-positive immune cells) and irradiated and *F. prausnitzii*-treated animals or by (d) IgG (negative control) in control animals. Scale bars 200 µm. Ctrl = Controls, IRR = Irradiated, Fprau = *F. prausnitzii*.

### Prophylactic F. prausnitzii results in a protection of a pool of crypt epithelial progenitor/stem cells and differentiated epithelial tuft cells from colorectal irradiation

**Figure 5.**
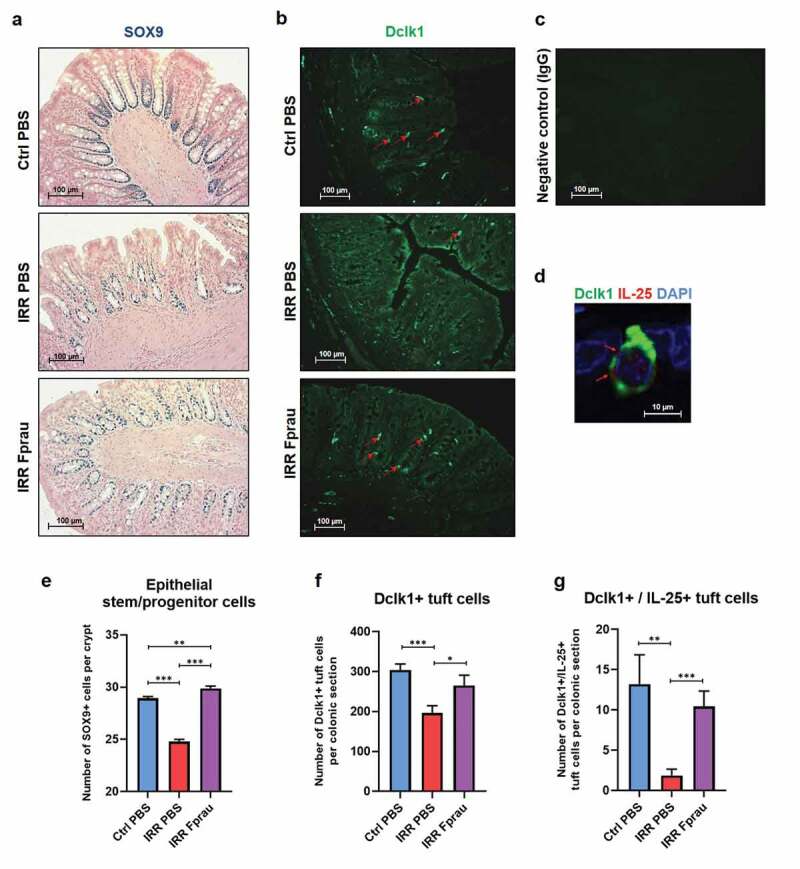
Effect of prophylactic *F. prausnitzii* treatment on 29 Gy colorectal irradiation-induced reduction of stem/progenitor cells and tuft cells at 3 d.

We assessed the number of epithelial stem/progenitor cells on colon histology slides stained by Sox-9 antibody (Figure 5(a), 5(e)). The number of Sox-9-positive stem/progenitor cells per crypt had decreased by 14.30%, 3 d after colorectal irradiation (28.93 ± 0.15 in control rats *vs* 24.79 ± 0.21 in irradiated rats, *p* < .001). In irradiated rats receiving prophylactic *F. prausnitzii* treatment, the number of Sox-9-positive cells stayed close to control level, with a slight but significant increase (29.87 ± 0.23 in irradiated and *F. prausnitzii* treatment *vs* 28.93 ± 0.15 in control rats, *p* < .01). This demonstrated that *F. prausnitzii* pre-treatment prevented radiation-induced loss of crypt epithelial stem/progenitor cells. We then investigated the ability of *F. prausnitzii* to preserve epithelial differentiated cells after colorectal irradiation. We focused on tuft cells, which are identified as an anatomically and functionally distinct epithelial cell entity. They are key regulatory intestinal niche cells and may well constitute one of the niche components.^18^ We measured the number of tuft cells on colon histology slides stained by Dclk1 antibody (Figure 5(b), 5(f)). In the colonic mucosa of control rats, there were an estimated 303.90 ± 15.10 Dclk1-positive tuft cells per slide. Three d after colorectal irradiation, the number of Dclk1-positive cells was only 195.7 ± 18.88, corresponding to a decrease of 35.6% of tuft cells (*p* < .001), and remained at control level in rats pretreated with *F. prausnitzii* (264.90 ± 26.10). This shows that prophylactic *F. prausnitzii* treatment also results in a protection of colonic-differentiated Dclk1-positive tuft cells from colorectal irradiation. After luminal infection by helminth parasites, cytokine IL-25 secreted by tuft cells stimulates stem/progenitor cells to regulate epithelial remodeling.^19,20^ We then performed double staining of colon histology slides by Dclk1 and IL-25 antibody (Figure 5(d), 5(g)). In control rats, we found that among 303.90 ± 67.53 Dclk1-positive tuft cells per slide only 13.20 ± 3.65 produce cytokine IL-25. Three days after colorectal irradiation, co-staining by Dclk1 and IL-25 was observed in 1.87 ± 0.77 tuft cells per slide, corresponding to an 85.80% decrease compared to controls (*p* < .01). In irradiated rats pretreated with *F. prausnitzii*, the number of tuft cells co-stained by Dclk1 and IL-25 remained at basal level (10.41 ± 1.92). *F. prausnitzii* also preserved the basal level of differentiated tuft cells that could provide an optimal microenvironment for stem cell function.

Representative pictures of (a) immunohistochemistry staining of colonic sections by SOX9 antibody (blue) and nuclear fast red counterstain (pink) for the quantification of crypt stem/progenitor cells (scale bars 100 µm), (b) immunofluorescence staining of colonic sections by Dclk1 antibody (in green) for the quantification of tuft cells (scale bars 100 µm), (c) immunofluorescence staining of colonic sections by IgG for negative control of DclK1 (scale bare 100 µm) and (d) immunofluorescence double-staining of colonic sections by Dclk1 and IL25 antibodies (in green and red, respectively, arrows show co-staining, Scale bar 10 µm). Sections show control animals, irradiated animals, and irradiated and *F. prausnitzii*-treated animals for (a-b) and control animals for (c-d). Histograms representing (e) the number of SOX9-positive cells per crypt, (f) the number of Dclk1-positive tuft cells per colonic section, and (g) the number of Dclk1- and IL-25-positive tuft cells per colonic section. Histogram (e) represents the average of 50–60 independent measurements per animal (n = 5 animals per group, N = 1), histograms (f) and (g) represent the average of 8 independent measurements per animals (5 animals per group (N = 1)). Ctrl = Controls, IRR = Irradiated, Fprau = *F. prausnitzii*. Error bars represent SEM, *p <.05, **p <.01, ***p <.001.

### Prophylactic F. prausnitzii treatment results in maintaining the self-renewal of colonic epithelium exposed to radiation and reduces mucosal ulceration

**Figure 6.**
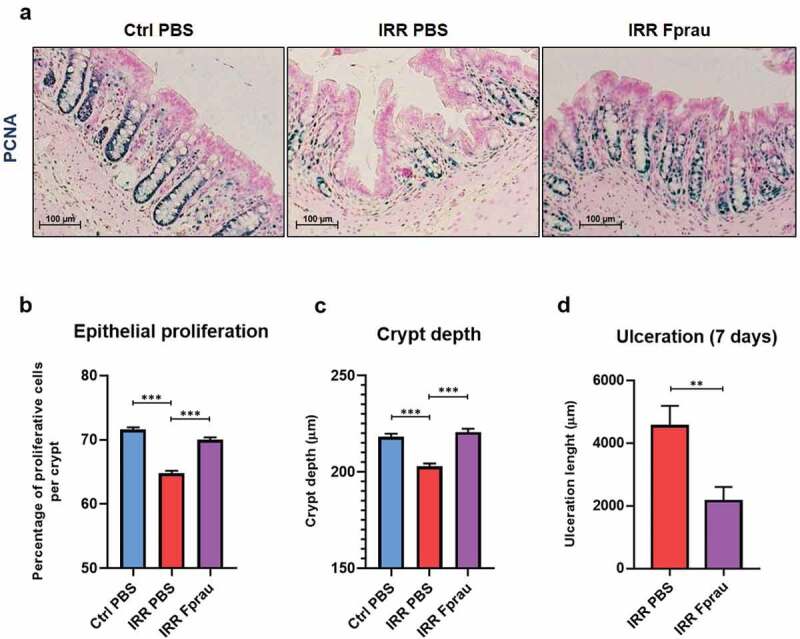
Effect of prophylactic *F. prausnitzii* treatment on 29 Gy colorectal irradiation-induced decrease of colonic mucosa renewal at 3 d and ulceration at 7 d.

We were then interested by the renewal ability of colonic epithelium, enhancement, or maintenance of which in response to exogenous stressors is key to physical barrier integrity. We first studied whether *F. prausnitzii-*induced protection of crypt epithelial stem/progenitor cells and tuft cells in irradiated rats was also associated with the ability of crypt epithelial cells to proliferate. We analyzed the number of proliferating crypt epithelial cells on histology colon slides stained by PCNA antibody (Figure 6(a-b)). The proliferative rate of crypt epithelial cells fell from 71.60 ± 0.31% of proliferative PCNA-positive cells per crypt in control rats to 64.70 ± 0.39% in irradiated rats at 3 d (*p* < .001). *F. prausnitzii* pre-treatment stabilized at basal level the proliferation of the entire array of crypt epithelial cells, including crypt stem/progenitor cells. We then measured the crypt depth, which in the colon is a criterion for mucosal thickness (Figure 6(c)). At 3 d, colorectal irradiation-induced partial atrophy of the crypt (7.02% reduction of crypt depth, 202.90 ± 1.51 µm in irradiated rats *vs* 218.24 ± 1.58 µm in control rats, *p* < .001). Pre-treatment of irradiated rats with *F. prausnitzii* maintained crypt depth at control level (220.70 ± 1.72 µm in irradiated and *F. prausnitzii* pre-treated rats *vs* 218.24 ± 1.58 µm in control rats). These results demonstrate the ability of *F. prausnitzii* pre-treatment to preserve the basal level of epithelial self-renewal even if rats are exposed to colorectal irradiation and support its acute effects on the preservation of colonic epithelial barrier integrity. The benefit provided by *F. prausnitzii* pre-treatment was sustained until 7 d after colorectal irradiation (Figure 6(d), Supplementary materials and methods). Prophylactic *F. prausnitzii* treatment reduced colorectal irradiation-induced mucosal ulceration by 52.80% at 7 d (4546.50 ± 650.08 µm in irradiated rats *vs* 2146.70 ± 458.50 µm in irradiated rats treated with *F. prausnitzii*; *p < .01).

(a) Representative pictures of immunohistochemistry staining of colonic sections by PCNA antibody (in blue) and nuclear fast red counterstain (in pink) for the quantification of crypt epithelial proliferation in control animals, irradiated animals, and irradiated and *F. prausnitzii*-treated animals. Scale bars 100 µm. (b) Histogram representing the percentage of proliferative PCNA-positive cells per crypt of the colon. Histograms representing quantitative analysis (c) of colonic crypt depth (µm) in each group at 3 d and (d) length of mucosal ulceration (µm) in irradiated rats and irradiated and *F. prausnitzii*-treated rats at 7 d, colonic sections were stained with hematoxylin, eosin and saffron (HES). For b and c, each value of the histogram represents the average of 50–60 independent measurements per animal (n = 5 animals per group, N = 1). For d, each value corresponds to the average of 20 independent measurements per animal (n = 8 animals per group, N = 1). Error bars represent SEM, **p <.01, ***p <.001 Ctrl = Controls, IRR = Irradiated, Fprau = *F. prausnitzii*.

## Discussion

This study demonstrated that prophylactic treatment with *F. prausnitzii* induces an acute protection of the colonic mucosal barrier from radiation exposure and therefore maintains its functional integrity. The therapeutic benefit of *F. prausnitzii* after irradiation is provided by its ability to prevent the increase of paracellular permeability, to enhance epithelial self-renewal and to preserve differentiated epithelial cells. The maintenance of colonic barrier integrity by *F. prausnitzii* is associated with macrophage recruitment and cytokine production by mucosal cells, mostly epithelial cells, as shown specifically for IL-18. We also found that *F. prausnitzii* treatment results in the preservation of colonic Dclk1/IL25-positive tuft cells.

Colorectal irradiation led to enhancement of colonic paracellular permeability and to neutrophil infiltration in the mucosa, which are indexes of mucosal barrier breakdown. We also quantified in the colonic mucosa a significant increase of the chemokine GRO/KC, a specific neutrophil chemoattractant, which is suggestive of a perpetuating cycle of neutrophil recruitment. We also showed in the mucosa a significant increase of macrophage inflammatory protein 3α (MIP3α), which is provided mostly by epithelial cells, but also by other cells such as innate and adaptative immune cells.^17,21,22^ Given that MIP3α is co-expressed with its physiological receptor CCR6 in neutrophils and that CCR6 expression is increased in activated neutrophils,^23^ the possibility of autocrine and/or paracrine signaling involving MIP3α, which increases neutrophil recruitment within colonic mucosa^23^ can also not be excluded 3 d after colorectal irradiation. Moreover, by its antimicrobial activity,^24^ increased secretion of MIP3α by other mucosal cells may, in synergy with neutrophil recruitment and activation, help protect against bacterial invasion. Three days after irradiation, we noted no modification of the production by mucosal cells of pro-inflammatory cytokines, and even quantified a significant decrease in the chemokine RANTES. Expression of RANTES is increased in activated epithelial cells, endothelial cells, fibroblasts, monocytes/macrophages within hours of stimulation^25^ and is induced late (3–5 d) after T lymphocyte activation.^26^ A colorectal irradiation-induced decrease in the expression of RANTES may be due to the radiosensitivity of many mucosal cell types leading to the deregulation of their molecular machinery and/or death and then to malfunction of the local immune response depending on monocyte/macrophages and T lymphocyte activation/and/or mobilization.^27–31^ Finally, under our experimental conditions, 3 d after colorectal irradiation seems to be too short a time to identify a shift in fecal microbial composition. Indeed, Gerassy-Vainberg et al.^32^ showed that targeted rectal radiation-induced time-dependent modification of fecal microbiota composition and that the radiation-induced unique microbial signature was most evident at 6 weeks. In conclusion, our results seem to be consistent with a nonspecific innate inflammatory response (neutrophil infiltration and activation) to colorectal irradiation at 3 d, with mostly phagocytic and antimicrobial effects.

In this context of radiation exposure, prophylactic treatment with *F. prausnitzii* had a protective effect on impairment of the colonic mucosal barrier. We first demonstrated that oral administration of *F. prausnitzii* prevented radiation-induced increase in acute (3 d) para-cellular permeability and in the nonspecific innate inflammatory response orchestrated by neutrophils after their mobilization (reduction of the number of neutrophils and GRO/KC expression in the colonic mucosa). The protective effect of *F. prausnitzii* on hyperpermeability has already been shown in a model of inflammatory bowel disease with chronic low-grade inflammation.^9,10^ In rodents receiving *F. prausnitzii* treatment for management of acute colitis, histological examination of the colon showed, as we quantified in our study in the colonic mucosa, a relatively low level of neutrophil infiltration.^7^ *F. prausnitzii* also reduced MPO activity in a model of inflammatory bowel disease with chronic mild/severe grade colitis,^33^ thus highlighting the ability of *F. prausnitzii* to reduce neutrophil activation in this context. Results obtained by Sokol et al. demonstrated an ability of *F. prausnitzii* supernatant (SN) to reduce *in vitro* IL-1β-induced IL-8 secretion by colonic epithelial cells (caco-2 cells).^6^ As IL-8 is known to be involved in neutrophil recruitment, the findings of Sokol et al. support our results and also suggest the involvement of epithelial activation in *F. prausnitzii* metabolite effects.

We then demonstrated that prophylactic treatment with *F. prausnitzii* also decreased epithelial damage at 3 d, leading to a reduced area of mucosal ulceration at 7 d. In our experimental conditions, *F. prausnitzii* treatment was unable to minimize radiation-induced apoptosis of crypt epithelial cells in the colonic mucosa (Supplementary data S2). Nevertheless, we showed for the first time that its therapeutic benefit at 3 d is related to its ability to preserve mucosal self-renewal through the maintenance of an activated pool of stem/progenitor cells and of epithelial cell proliferation.

This work supports and offers some assumptions concerning *F. prausnitzii* mechanisms of action probably through an increase in some inflammatory parameters.

In several experiments using models of inflammatory bowel disease, one mechanism proposed to explain the therapeutic benefits of *F. prausnitzii* is its anti-inflammatory properties.^6,7,12^ In our experimental context, in which colorectal irradiation-induced at 3 d a nonspecific immune response without a change in local pro-inflammatory cytokine production, we noted no anti-inflammatory effects after *F. prausnitzii* administration. Indeed, mucosal anti-inflammatory cytokine IL-10 was similar in control and irradiated animals pre-treated or not by *F. prausnitzii*.

Nevertheless, we showed that in rats receiving *F. prausnitzii*, irradiation led to significant enhancement of resident macrophages. Macrophages play a key role in coordinating signals from luminal microbiota and injured colonic epithelium and thereby transmit regenerative inputs to colonic progenitor cells.^34,35^ Recently, macrophages have been reported to be essential for the regenerative response of the intestine to lethal abdominal irradiation by rescuing LGR5+ intestinal stem cells.^35^ In the context of radiation-induced dysregulation of colonic epithelial self-renewal, an increase of resident macrophages by *F. prausnitzii* treatment may play a role in the protection of the epithelial progenitor/stem cell compartment and thus may help maintain colonic barrier integrity.

Moreover, in rats receiving *F. prausnitzii*, irradiation also induced up-regulation of the pro-inflammatory cytokines IL-1β, IL-18 by colonic mucosal cells. In a context in which the luminal level of *F. prausnitzii* could be increased, cell stress induced by irradiation should provide increased danger signals, which may stimulate NOD-like receptor (NLR) inflammasome signaling, leading to IL-1β and IL-18 activation and production by mucosal cells. It was previously reported that NLR inflammasome activation may be either regenerative or deleterious, depending on the cell type in which it is activated.^36^ In response to colitis, acute or chronic NLR inflammasome activation in colonic epithelial cells and the subsequent production and secretion of IL-18 seem to be critical for maintaining microbiota stability, colonic homeostasis/regeneration, and barrier integrity against bacterial translocation,^37,38^ whereas chronic NLR activation in immune cells like macrophages seems to have a pro-inflammatory effect.^36^ The severity of colonic injury may be modulated by shifting the balance between protective and damaging effects depending on the inflammatory context and the experimental conditions. In our experimental conditions, we found that *F. prausnitzii* treatment after irradiation increases IL-18 production mostly by epithelial cells. So, in this context, the balance between regenerative and deleterious effects is probably in favor of protection via epithelial cell activation. Further experiments are needed to assess the relative impact of IL-18 produced by crypt epithelial cells in the protective effect of *F. prausnitzii*.

Finally, *F. prausnitzii* effects are also associated with its ability to preserve differentiated epithelial cells like Dclk1-positive tuft cells from radiation-induced loss. The use of Villin-Cre; Dclk1^flox/flox^ mice, which are specifically deficient in Dclk1 in the epithelial cells of the intestine, showed the involvement of this enzyme in the regeneration of the epithelium of the small intestine after whole-body irradiation. Moreover, Dclk1 mainly produced by the tuft cells in the epithelium seems to have a radioprotective effect on stem/progenitor cells. In addition, in the absence of Dclk1 expression by tuft cells, irradiation-surviving stem/progenitor cells appear to lose their ability to provide epithelial repair and Dclk1 deletion seems to have an impact on animal survival.^39,40^ In the present study, the preservation of Dclk1-positive tuft cells by *F. prausnitzii* administration may have protected the colonic mucosa from radiation-induced damage. As chemosensory and secretory cells, tuft cells orchestrate the sensing of luminal content, the stromal immune response and regeneration of intestinal epithelial cells through the secretion of IL-25.^19,20,41^ We showed that some tuft cells quantified in control rats or after irradiation in rats treated with *F. prausnitzii* also expressed IL-25 cytokine, whereas after irradiation alone they did not or did so rarely. This result points to the potential involvement of tuft cells in the prophylactic effects of *F. prausnitzii* on colonic mucosa self-renewal, through Dclk1 and/or IL-25 production. Further experiments are needed to demonstrate robustly the role played by tuft cells in the efficacy of *F. prausnitzii* against radiation toxicity.

Finally, in order to further our understanding with respect to the mechanisms of action of *F.prausnitzii*, we analyzed the microbiota composition through 16S rRNA sequencing. No effect of irradiation or *F.prausnitzii* administration was detected on microbiota richness (α-diversity). A deeper look into our results showed that only few rats, 4 from 48, were positive for *F.prausnitzii* and moreover, they were distributed among all the groups tested (control group, irradiated group and irradiated and *F.prausnitzii* treated group, data not shown). We also performed qPCR specific from *F.prausnitzii* but no amplifications were detected for any group using this method (data not shown). *F.prausnitzii* is Extremely Oxygen Sensitive and also very sensitive to pH^42^ meaning that oral administration might lead to a massive death of the bacteria. We have already shown that it is indeed very difficult to colonize even germ-free mice with *F.prausnitzii*.^43^ Rodents do not exhibit the same level of endogenous *F.prausnitzii* compared to humans which is one of the main difference between the two microbiota.^44^ We have thus no evidence of *F.prausnitzii* colonization or even persistence in rats (this study) or in mice (chatel, personal communication) but there is still a protective effect for some parameters indicating that implementation is not required or it is at an undetectable level.

In a context of radiation-induced colonic damage, we have demonstrated for the first time that prophylactic *F. prausnitzii* treatment favors the reinforcement of the colonic mucosal barrier leading to radioprotective effects. We have established that *F. prausnitzii* prevents radiation-induced para-permeability and the associated acute nonspecific immune response, but also preserves epithelial integrity. Our results suggest that epithelial cell activation is involved in the benefits of acute *F. prausnitzii* treatment and that tuft cells could play a key role. Our study opens new perspectives in the protection of colonic mucosa from radiation toxicity using prophylactic *F. prausnitzii* treatment. New-generation probiotics might be a real hope in reducing acute side effects in patients who undergo pelvic radiotherapy. It has also been suggested that the severity of acute radiation toxicity may predict the severity of chronic symptoms, known as consequential late damage.^45^ Therefore, *F. prausnitzii*-induced acute protection may be beneficial in reducing late side effects. Clinical use of *F. prausnitzii* could help improve the quality of life of these patients.

## Supplementary Material

Supplemental Material
