# Supplementary material for: Prophylactic Faecalibacterium prausnitzii treatment prevents the acute breakdown of colonic epithelial barrier in a preclinical model of pelvic radiation disease
Source: Gut Microbes. 2020 Sep 28;12(1):1812867. doi: 10.1080/19490976.2020.1812867 (PMC7524396; doi:10.1080/19490976.2020.1812867)
Supplement: Supplemental Material [file KGMI_A_1812867_SM2512.zip › Supplementary information/Supplementary data 1.docm]

S**1: Effect of prophylactic *F. prausnitzii* treatment on chemokine/cytokine production by colonic mucosal cells 3 days after 29 Gy colorectal irradiation.**


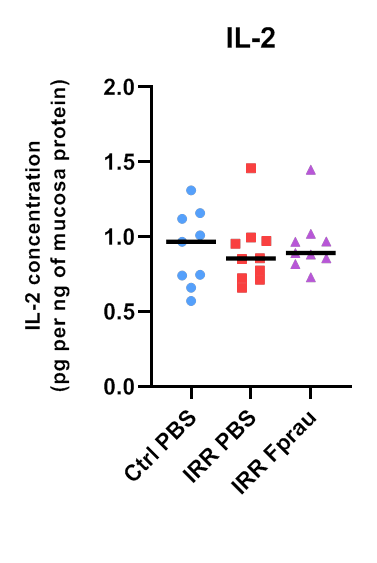

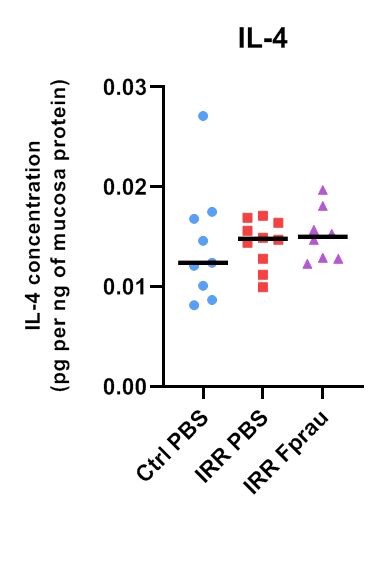

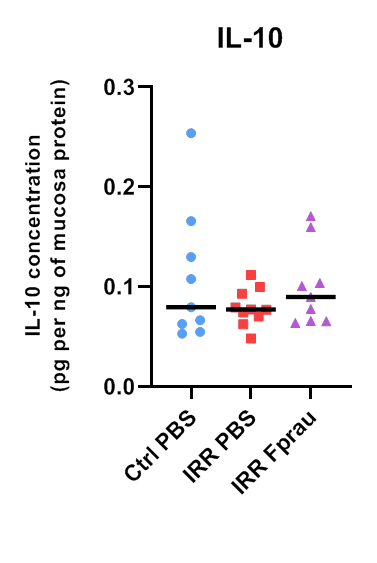

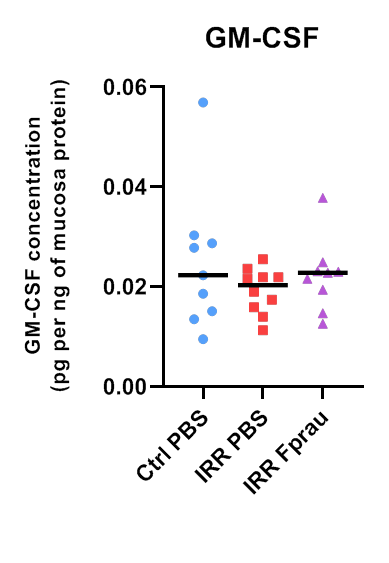

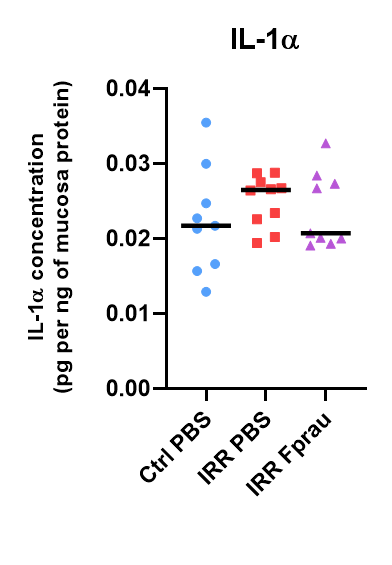

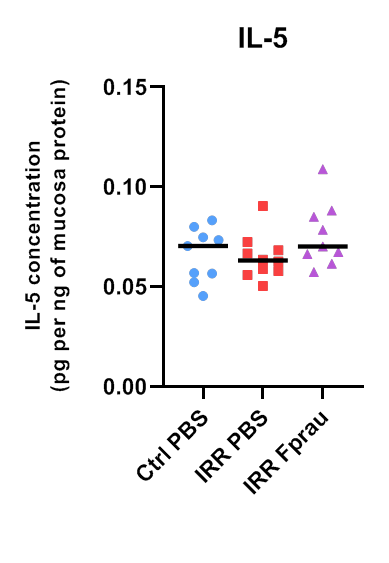

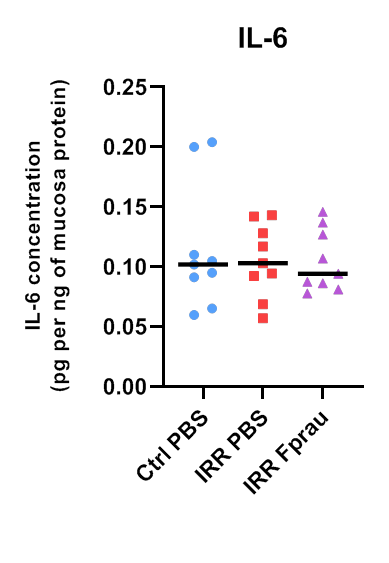

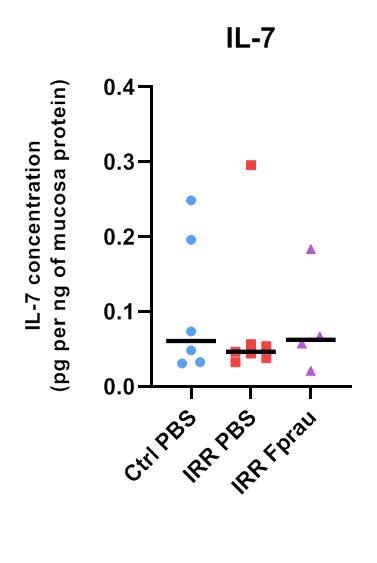

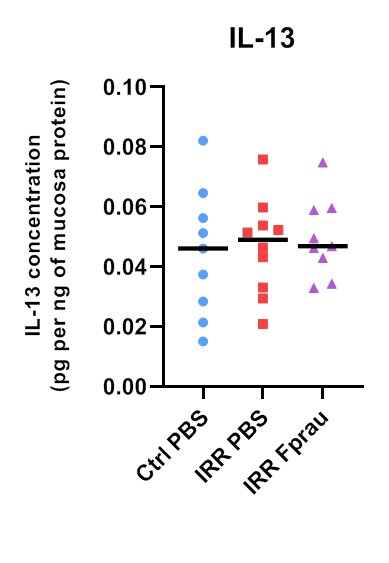

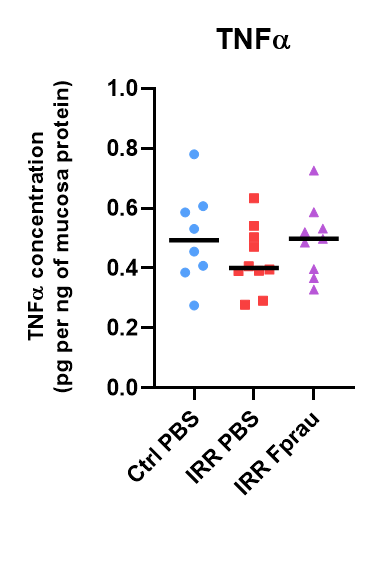

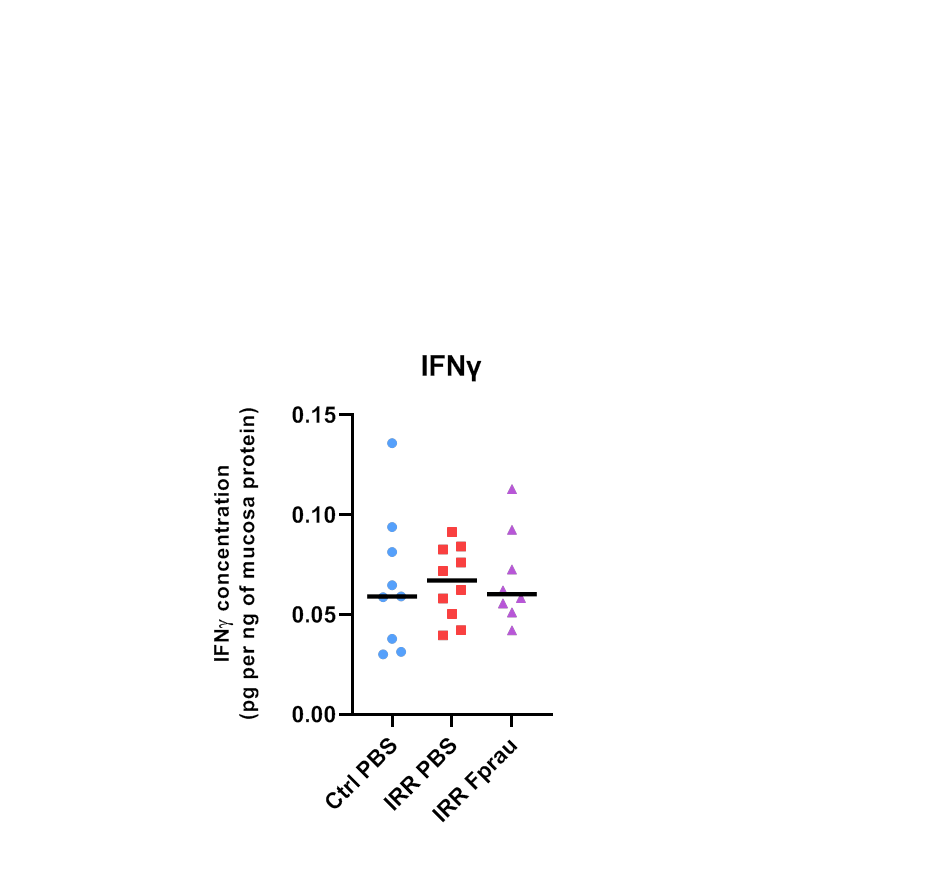

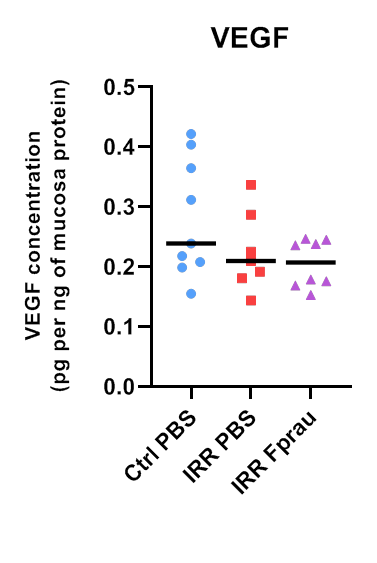

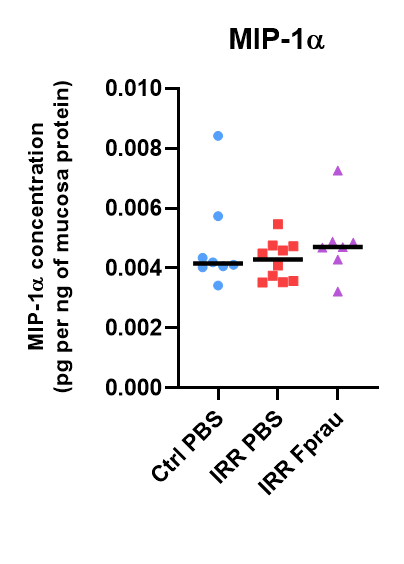

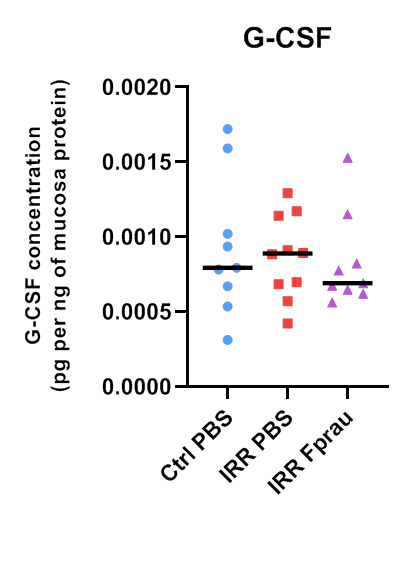


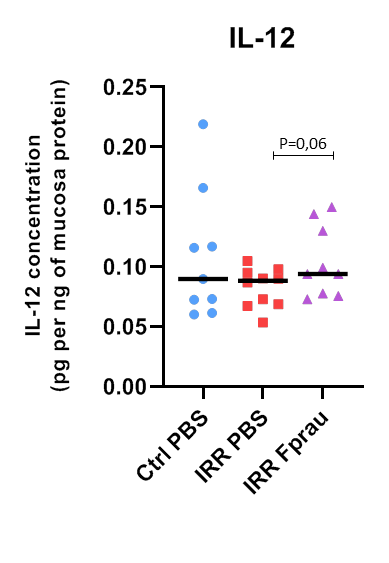


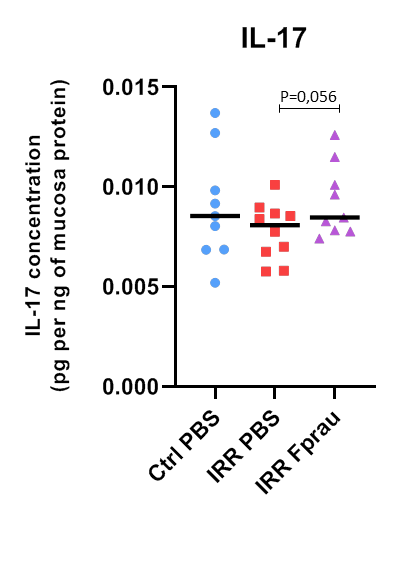


**S1. Effect of prophylactic *F. prausnitzii* treatment on chemokine/cytokine production by colonic mucosal cells 3 days after 29 Gy colorectal irradiation.** The graphs represent the expression in the colonic mucosa of the cytokines IL-1α, IL-2, IL-4, IL-6, IL-7, IL-10, IL-12, IL-13, IL-17, TNFα, IFNγ and VEGF, and of the chemokines MIP-1α, GM-CSF and G-CSF in control animals, irradiated animals or irradiated and *F. prausnitzii*-treated animals. ELISA multiplex assays were performed at least in duplicate per animal and 9 to 10 animals were used per group (N=1). Ctrl= Controls, IRR=Irradiated, Fprau=*F. prausnitzii*. No significant variation of cytokine/chemokine expression in the colonic mucosa was reported.
