# Supplementary material for: Prophylactic Faecalibacterium prausnitzii treatment prevents the acute breakdown of colonic epithelial barrier in a preclinical model of pelvic radiation disease
Source: Gut Microbes. 2020 Sep 28;12(1):1812867. doi: 10.1080/19490976.2020.1812867 (PMC7524396; doi:10.1080/19490976.2020.1812867)
Supplement: Supplemental Material [file KGMI_A_1812867_SM2512.zip › Supplementary information/Supplementary data 2.docm]

**S2: Effect of prophylactic *F. prausnitzii* treatment on 29 Gy radiation-induced colonic cell apoptosis at 6 hours.**

We studied crypt epithelial cells in apoptosis on colon histology slides by TUNEL assay. In control rats, there were an estimated 5.00 ± 0.50 TUNEL-positive apoptotic epithelial cells per crypt. The number of apoptotic epithelial cells per crypt had increased to 252.00 ± 23.00 six hours after colorectal irradiation (p< .001). *F. prausnitzii* pre-treatment did not avoid radiation-induced crypt epithelial cell apoptosis. Three days after colorectal irradiation, the level of crypt epithelial cells in apoptosis returned to control level (data not shown).


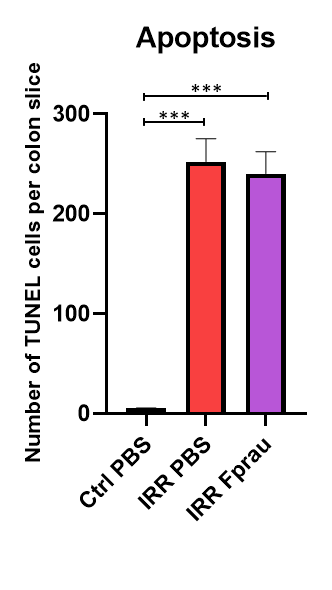


**S2. Effect of prophylactic *F. prausnitzii* treatment on radiation-induced colonic cell apoptosis at 6 hours.** The histogram represents the number of crypt apoptotic TUNEL-positive cells per colonic section. The value of apoptosis represents the average of 50-60 independent measurement per animal (n=5 animals per group, 20 independent colonic sections per rats were analyzed, N=1). Ctrl=Controls, IRR=Irradiated, Fprau=*F. prausnitzii*. Error bars represent S.E.M, ***p< .001
