# Supplementary material for: Prophylactic Faecalibacterium prausnitzii treatment prevents the acute breakdown of colonic epithelial barrier in a preclinical model of pelvic radiation disease
Source: Gut Microbes. 2020 Sep 28;12(1):1812867. doi: 10.1080/19490976.2020.1812867 (PMC7524396; doi:10.1080/19490976.2020.1812867)
Supplement: Supplemental Material [file KGMI_A_1812867_SM2512.zip › Supplementary information/Supplementary Materials and Methods.docm]

**Supplementary materials and methods**

**Protocol of *F. prausnitzii* administration (at 7 days)**

The irradiation protocol was similar to those used previously. Seven days before irradiation, 1.10^9^ CFU of bacteria or PBS was administered intragastrically each day, and then daily until three days after irradiation.

**Measurement of ulceration length on histology slides (at 7 days)**

Animals were sacrificed by isoflurane inhalation 7 days after colorectal irradiation. Distal colons were removed, longitudinally cut along the mesentery, fixed in 4% formaldehyde and embedded in paraffin. Paraffin-embedded colons were cut on a rotary microtome (Leica Microsystems AG, Wetzlar, Germany) into serial longitudinal sections of 5 µm, in 5 series spaced by 250 µm and stained with hematoxylin-eosin-saffron (HES). The length (µm) of colorectal damage was assessed by measuring with Histolab software (Microvision Instruments, Lisses, France).
